# Construct and Predictive Validity of the Kansas City Cardiomyopathy Questionnaire in Adult Congenital Heart Disease

**DOI:** 10.1016/j.jacadv.2025.102020

**Published:** 2025-07-24

**Authors:** Ryan D. Byrne, Ajith P. Nair, Stephen J. Dolgner, Swati Choudhry, Edward J. Hickey, Alexis C. Wood, Savitri Fedson, Biykem Bozkurt, Christopher R. Broda

**Affiliations:** a Adult Congenital Heart Program, Department of Pediatrics, Section of Cardiology, Texas Children's Hospital, Baylor College of Medicine, Houston, Texas, USA; b Division of Cardiothoracic Transplantation and Circulatory Support, Michael E. DeBakey Department of Surgery, Baylor College of Medicine, Houston, Texas, USA; c Department of Cardiopulmonary Transplantation and the Center for Cardiac Support, Texas Heart Institute, Houston, Texas, USA; d Department of Pediatrics, Section of Cardiology, Baylor College of Medicine/Texas Children’s Hospital, Houston, Texas, USA; e Division of Congenital Heart Surgery, Michael E. DeBakey Department of Surgery, Baylor College of Medicine and Texas Children’s Hospital, Houston, Texas, USA; f USDA/ARS Children’s Nutrition Research Center, Baylor College of Medicine, Houston, Texas, USA; g Center for Medical Ethics and Health Policy, Baylor College of Medicine, Houston, Texas, USA; h Department of Medicine, Michael E. DeBakey VA Medical Center, Houston, Texas, USA; i Winters Center for Heart Failure Research, Cardiovascular Research Institute, Baylor College of Medicine, DeBakey VA Medical Center, Houston, Texas, USA

**Keywords:** adult congenital heart disease, construct validity, heart failure, Kansas City Cardiomyopathy Questionnaire, predictive validity

## Abstract

**Background:**

The Kansas City Cardiomyopathy Questionnaire (KCCQ) is an Food and Drug Administration-approved health status measure for patients with heart failure (HF) but has not been directly assessed in adult congenital heart disease (ACHD).

**Objectives:**

This study evaluates construct and predictive validity of the KCCQ in ambulatory ACHD patients.

**Methods:**

We performed cross-sectional and prospective cohort studies of ACHD patients who completed the KCCQ to assess construct and predictive validity, respectively. KCCQ scores were compared across ACHD complexity categories, disease characteristics and physiologic failure, and within 2 primary composite outcomes: death or all-cause hospitalization (DH) and death or interventional cardiac procedures (DP). Survival analysis was performed for each composite outcome.

**Results:**

A total of 109 patients completed 129 KCCQs (median age 29.0 years; 41.3% female). Median KCCQ scores were significantly lower for anatomically complex patients (76.6 in great complexity, 89.0 in simple/moderate complexity; *P* = 0.015), advanced ACHD physiological stage (72.6 in D, 98.0 in A; *P* < 0.001), patients with physician-reported HF (72.2 vs 87.2 without HF; *P* < 0.001), and advanced NYHA functional class (NYHA III: 44.4, NYHA I: 96.5; *P* < 0.001). Median follow-up time was 16.3 months in the predictive validity analysis (N = 92 patients). KCCQ was significantly lower for ACHD patients who experienced DH (66.0 vs 85.0; *P* = 0.002) or DP (53.5 vs 85.0; *P* = 0.001). Patients scoring ≤50 had significantly worse event-free survival (*P* = 0.004 for DH; *P* = 0.001 for DP).

**Conclusions:**

The KCCQ has construct and predictive utility in ACHD and provides an opportunity to assess health status in ACHD both clinically and in future ACHD research.

Heart failure (HF) is the leading cause of death for adults with congenital heart disease (ACHD). The incidence of ACHD HF is rising^1^ and is particularly more common in patients with the most severe congenital heart disease including systemic right ventricle and the Fontan population.^2^ Within ACHD, earlier identification of HF symptoms and timely referral for advanced therapy consideration, including transplant evaluation, has been associated with increased likelihood of transplant listing^1^ and survival following transplantation.^3^

In this context, tools such as patient-reported outcomes (PROs) that can accurately capture the extent of disease-related symptomatology, quality of life, and health status in patients with ACHD HF are of substantial importance. The diverse spectrum of anatomic and physiologic substrates seen in ACHD and ACHD HF has led investigators to speculate that an ACHD-specific PRO tool is needed.^4^ However, despite the mechanistic heterogeneity of ACHD HF^5^, many patients with ACHD experience similar symptoms once advanced disease is manifest^6^, due to shared mechanisms such as decreased cardiac output, increased left atrial pressures, and/or cyanosis. Overlapping symptom experience provides an opportunity to utilize well established PROs designed for HF in the non-ACHD population, such as the Kansas City Cardiomyopathy Questionnaire (KCCQ).

The KCCQ is Food and Drug Administration approved for Clinical Outcome Assessment^7^ and is a validated health status measure for patients with HF^8^ in both reduced and preserved ejection fraction phenotypes.^9^ Its multiple utilities include routine clinical care, as an outcome in clinical trials,^10^, ^11^, ^12^ and in quality assessment.^13^ The KCCQ has 23 items that cover 7 domains of clinical interest. Scores are represented on a scale of 0 to 100 with lower scores signifying a worse health status.^14^ In the non-ACHD ambulatory setting, the KCCQ has been associated with prognosticating patient outcome.^15^^,^^16^ While investigators may have previously utilized the KCCQ in a small ACHD HF study,^17^ it has yet to be validated in a cohort of ACHD patients. Thus, its relevance within this population remains unclear.

We believe the KCCQ has utility in the ACHD population as a health status measure and have accordingly constructed a study to validate this important and pervasive PRO measure. We hypothesize that: 1) lower KCCQ scores will be associated with great anatomic complexity, advanced ACHD physiological stage, and other markers of health status such as NYHA functional class, thereby demonstrating construct validity within ACHD; and 2) KCCQ scores will be lower in ACHD patients who ultimately experience death, all-cause hospitalization, and interventional cardiac procedures, thereby demonstrating predictive validity.

## Methods

### Construct validity

For the construct validity portion of our analysis, we performed a single-center cross-sectional study of patients who completed the KCCQ electronically in our ACHD clinic from July 2021 to April 2023. Patients who completed the KCCQ who did not have a diagnosis of congenital heart disease (ie, patients with inherited cardiomyopathy or referrals for symptoms such as chest pain or palpitations in the absence of congenital heart disease) were excluded. If a patient submitted additional KCCQ surveys in subsequent visits, only the first KCCQ completed was included. Patient and disease demographics were collected including age, sex, cardiac diagnosis, and cardiac comorbidities such as atrial or ventricular arrhythmia, need for permanent pacemaker, cyanosis, and heterotaxy syndrome. Atrial arrhythmia was defined as having a history of atrial tachycardia, atrial fibrillation, atrioventricular reentrant tachycardia, atrioventricular nodal reentrant tachycardia, and typical or atypical atrial flutter. Ventricular arrhythmia included history of ventricular tachycardia (including nonsustained episodes) or ventricular fibrillation. Premature atrial or ventricular contractions were not considered arrhythmia. Physician assessments of physiologic failure were obtained via clinical documentation. ACHD anatomic complexity and physiological stage were defined according to the American Heart Association/American College of Cardiology 2018 ACHD guidelines.^18^ NYHA functional class and reporting of HF as a patient diagnosis were determined by the ACHD physician. Echocardiographic data, including assessment of systemic ventricular systolic function and atrioventricular valve function, were included if obtained within 12 months of KCCQ completion. Echocardiographic evaluation was performed at the discretion of the cardiologist, including utilization of qualitative assessments, particularly when considering systolic function of a systemic right ventricle.

### Predictive validity

To assess for predictive validity of the KCCQ in ACHD, a prospective cohort study was performed utilizing the same single-center cohort as in our construct validity analysis, however, patients with less than 6 months of follow-up after completion of the KCCQ were excluded in this portion of our study. KCCQ scores were categorized into the following score groups: 76 to 100, 51 to 75, and 0 to 50. Due to the relatively low number of patients in our cohort who scored ≤50, our lowermost score group combines the bottom 2 traditional KCCQ score quartiles frequently utilized in the literature.^14^^,^^15^

We defined 2 primary outcome variables for the predictive validity analysis. The first is a composite of death or all-cause hospitalization (DH). Recognizing the heterogenic etiology of physiologic failure in ACHD, we defined the second outcome variable as a composite of death or interventional cardiac procedures (DP) including cardiac surgery, cardiac catheterization intervention, electrophysiological ablation, or placement of a permanent pacemaker. Diagnostic cardiac catheterizations without an interventional component were not included.

### Statistical analysis

For construct validity, demographic comparisons within the overall cohort were stratified by anatomic complexity. For this analysis, simple and moderate anatomic complexity patients were grouped together given the low number of patients with simple anatomic complexity. Chi-square test was used for categorical predictor variables and Mann-Whitney *U* test was used for continuous predictor variables. Predictor variables for the construct validity analysis included patient characteristics as well as assessments of physiologic failure. KCCQ score was the outcome variable. Mann-Whitney *U* test and Spearman’s correlation were utilized when predictor variables were represented as binomial and ordinal categorical variables, respectively.

For predictive validity, patient demographics were stratified by the aforementioned KCCQ score groups. Chi-squared and Kruskal-Wallis tests were utilized for categorical and continuous demographic variables, respectively. KCCQ score was defined as the predictor variable and was compared between those who did and did not meet the outcomes of DH and DP using Mann-Whitney *U* test. To further test the prognostic ability of KCCQ in ACHD, Kaplan-Meier curves were constructed. Due to the presence of a nonproportional distribution, restricted mean survival time was used to compare event-free survival curves of each outcome variable stratified by KCCQ score group over a time period of 22 months. If a patient experienced 2 or more outcomes, the time at which the first outcome occurred was used for the survival analysis.

This study was approved by the Baylor College of Medicine Institutional Review Board. All statistical analysis was performed using Statistical Package for the Social Sciences (SPSS, SPSS Inc) version 29.0. The data underlying this article are available from the corresponding author upon reasonable request. License to utilize the KCCQ for the purpose of scholarly work was purchased via Outcomes Instruments, LLC.

## Results

### Construct validity

A total of 159 patients completed a KCCQ survey in our ACHD clinic. Fifty patients were excluded due to having a diagnosis other than ACHD, leaving 109 patients who completed 129 total KCCQ surveys. While the KCCQ was offered to all patients in our ACHD clinic, the majority (66.1%) were completed in our ACHD HF clinic. Patient demographics including NYHA functional class and ACHD physiological stage stratified by anatomic complexity are demonstrated in Table 1. A detailed list of cardiac diagnoses within each anatomic complexity category can be found in the Supplemental Appendix. The median age of the population was 29.0 years and the minority (41.3%) of patients were female. The majority of patients in the study had biventricular hearts (67.0%). Patients with single ventricle made up nearly one-third of the overall cohort (32.1%) and represented the slight majority of those with great complexity (51.5%). Within the overall cohort, 77 patients (70.6%) had a systemic left ventricle, 31 (28.4%) had a systemic right ventricle, and 1 patient (0.9%) had indeterminate ventricular morphology. Compared to those with simple or moderate complexity, patients with great anatomic complexity were significantly more likely to have atrial arrhythmia (54.4% vs 19.5%; *P* < 0.001) and significantly more likely to have a history of permanent pacemaker placement (35.3% vs 7.3%; *P* = 0.001). Ventricular arrhythmia in the total cohort was relatively common (29.4%), though no significant difference was detected across complexity groups. Compared to simple/moderate complexity, patients with great complexity were more likely to have a diagnosis of heterotaxy syndrome (11.8% vs 0.0%; *P* = 0.023) and have lower systemic oxygen saturations (94.0% vs 97.0%; *P* < 0.001). Average time from KCCQ to assessment by echocardiogram was 1.8 months. Only 20.2% of the overall cohort had systemic ventricular dysfunction that was reported as moderate or worse and the proportion of patients with ventricular dysfunction was similar between anatomic complexity groups. A significantly greater proportion of patients with moderate or worse atrioventricular valve dysfunction was identified in patients with great anatomic complexity compared to simple or moderate complexity (17.7% vs 2.4%; *P* = 0.026). Patients with great anatomic complexity were more likely to have worse NYHA functional class compared to simple/moderate complexity (*P* = 0.016), though notably, only one patient in the overall cohort was assessed as having NYHA IV. No statistically significant difference was detected between anatomic complexity groups when comparing ACHD physiological stage.

**Table 1:** Overall Patient and Disease Demographics Stratified by Anatomic Complexity

	Overall (N = 109)	Simple or Moderate Complexity (n = 41, 37.6%)	Great Complexity (n = 68, 62.4%)	*P* Value
Age (y)	29.0 (21.1-43.6)	27.1 (19.7-37.8)	32.9 (22.8-45.6)	0.051
Sex (female)	45 (41.3%)	20 (48.8%)	25 (36.8%)	0.217
Systemic ventricular morphology				
Biventricular left	55 (50.5%)	41 (100.0%)	14 (20.6%)	<0.001
Biventricular right	18 (16.5%)	0 (0.0%)	18 (26.5%)	
Single ventricle left	22 (20.2%)	0 (0.0%)	22 (32.4%)	
Single ventricle right	13 (11.9%)	0 (0.0%)	13 (19.1%)	
Indeterminate morphology	1 (0.9%)	0 (0.0%)	1 (1.4%)	
Atrial arrhythmia	45 (41.3%)	8 (19.5%)	37 (54.4%)	<0.001
Ventricular arrhythmia	32 (29.4%)	10 (24.4%)	22 (32.4%)	0.377
History of pacemaker	27 (24.8%)	3 (7.3%)	24 (35.3%)	0.001
Heterotaxy	8 (7.4%)	0 (0.0%)	8 (11.8%)	0.023
Systemic oxygen saturation (%)	96.0 (92.0-98.0)	97.0 (97.0-99.0)	94.0 (90.5-97.0)	<0.001
Systemic ventricular dysfunction ≥moderate	22 (20.2%)	5 (12.2%)	17 (25.0%)	0.165
Atrioventricular valve dysfunction ≥moderate	13 (11.9%)	1 (2.4%)	12 (17.7%)	0.026
NYHA functional class	(N = 89)	(n = 30, 33.7%)	(n = 59, 66.3%)	
I	26 (29.2%)	15 (50.0%)	11 (18.7%)	0.016
II	44 (49.5%)	12 (40.0%)	32 (54.2%)	
III	18 (20.2%)	3 (10.0%)	15 (25.4%)	
IV	1 (1.1%)	0 (0.0%)	1 (1.7%)	
ACHD physiological stage	(N = 89)	(n = 31, 34.8%)	(n = 58, 65.2%)	
A	4 (4.5%)	1 (3.2%)	3 (5.2%)	0.432
B	12 (13.5%)	6 (19.4%)	6 (10.3%)	
C	45 (50.5%)	17 (54.8%)	28 (48.3%)	
D	28 (31.5%)	7 (22.6%)	21 (36.2%)	

Values are n (%) or median (IQR).

Test used: chi-square test used for categorical variables and Mann-Whitney *U* test used for continuous variables.

ACHD = adult congenital heart disease.

Table 2 represents a comparison of KCCQ scores for patients with or without key patient characteristics and assessments of physiologic failure. Patients with systemic right ventricle scored significantly lower on the KCCQ than patients with a systemic left ventricle (65.4 vs 84.0; *P* = 0.016) while no significant difference in KCCQ score was detected in patients with single ventricle physiology compared to a biventricular circulation (75.6 vs 83.5; *P* = 0.670). Significantly lower KCCQ scores were identified for patients with atrial arrhythmia (69.8 vs 86.9; *P* = 0.002) and with a history of permanent pacemaker placement (64.6 vs 84.2; *P* = 0.002). History of ventricular arrhythmia was not associated with a significant difference in KCCQ score (82.7 vs 80.7; *P* = 0.992). Patients with systemic oxygen saturation <95% scored significantly lower on KCCQ compared to patients with higher oxygen saturations (75.1 vs 89.0; *P* = 0.013). Median KCCQ scores for patients with and without ventricular dysfunction or atrioventricular valve dysfunction were similar. Patients who were deemed to have HF by their ACHD physician had significantly lower KCCQ scores compared to those without HF (72.2 vs 87.2; *P* < 0.001). ACHD patients who were assessed to have NYHA III/IV symptoms scored significantly lower on the KCCQ compared to those with NYHA I/II symptoms (43.8 vs 88.1; *P* < 0.001). Patients with ACHD physiological stages of C or D also scored significantly lower compared to those with physiological stages A or B (78.8 vs 95.7; *P* < 0.001).

**Table 2:** Kansas City Cardiomyopathy Questionnaire Scores in Key ACHD Clinical Features and Assessments of Physiologic Failure

	Yes	No	*P* Value
Patient characteristics			
Systemic right ventricle	65.4 (51.1-89.7)	84.0 (65.8-96.0)	0.016
Single ventricle	75.6 (55.2-92.7)	83.5 (61.9-95.1)	0.670
Heterotaxy	73.5 (50.4-97.7)	81.0 (61.9-94.4)	0.958
Atrial arrhythmia	69.8 (51.5-85.0)	86.9 (67.3-96.5)	0.002
Ventricular arrhythmia	82.7 (54.2-94.4)	80.7 (59.4-94.9)	0.992
History of pacemaker	64.6 (42.4-89.6)	84.2 (65.3-95.8)	0.002
Noninvasive physiologic performance			
Systemic oxygen saturation <95%	75.1 (56.2-84.6)	89.0 (62.4-96.7)	0.013
Systemic ventricular dysfunction ≥moderate	76.7 (52.7-90.6)	83.6 (61.2-95.4)	0.284
Systemic atrioventricular valve dysfunction ≥moderate	81.0 (65.6-93.8)	81.0 (55.1-94.9)	0.327
Assessments of physiologic failure			
Physician-reported heart failure	72.2 (43.5-86.3)	87.2 (68.6-97.1)	<0.001
NYHA functional class III or IV	43.8 (28.3-76.5)	88.1 (70.0-96.7)	<0.001
ACHD physiological stage C or D	78.8 (50.6-93.4)	95.7 (84.1-98.9)	<0.001

Abbreviation as in Table 1.

Values are median (IQR).

Test used: Mann-Whitney *U* test.

A similar, domain-specific analysis was performed, the results of which are available in the Supplemental Appendix. In general, domain scores for symptom burden, symptom frequency, physical limitations, social limitations, and quality of life were correlative with the overall KCCQ score for each given patient characteristic, and no particular domain stood out as the predominant impetus for overall health status. Construct validity analyses were additionally performed with the inclusion of all completed KCCQ surveys (some patients represented more than once) and can be found in the Supplemental Appendix.

**Figure 1:**
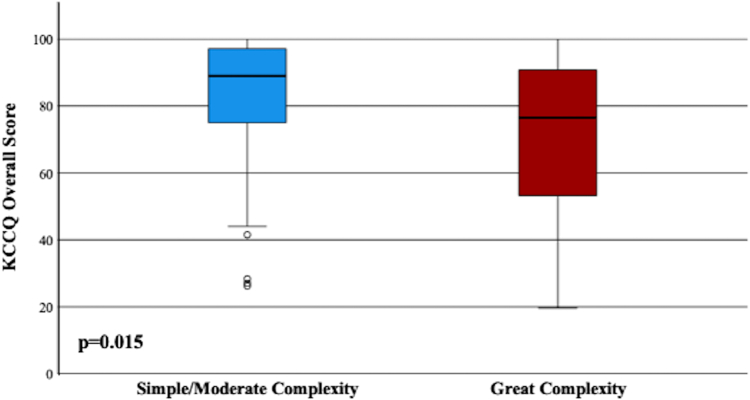
**KCCQ Scores Stratified by Anatomic Complexity Groups** Median KCCQ scores were compared across anatomic complexity groups. Compared to simple/moderate complexity (89.0), KCCQ scores were significantly lower for patients with great anatomic complexity (76.6). KCCQ = Kansas City Cardiomyopathy Questionnaire.

**Figure 2:**
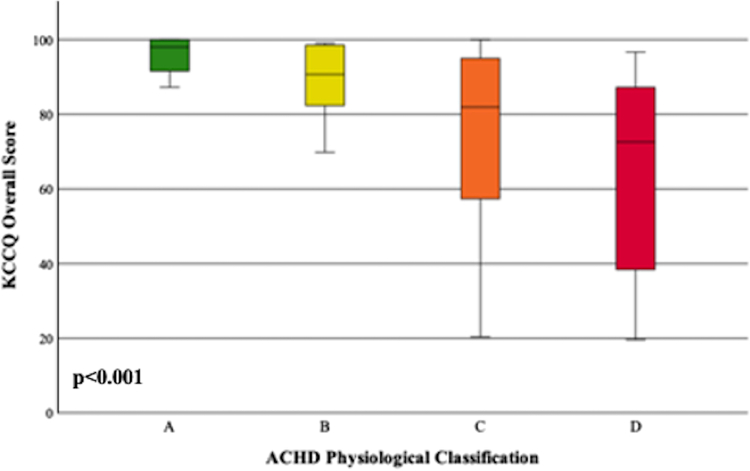
**KCCQ Scores Stratified by ACHD Physiological Stages** Median KCCQ scores were compared across ACHD physiological stages. Scores progressively declined with worsening of physiological stage: 98.0 in stage A, 90.7 in stage B, 81.9 in stage C, and 72.6 in stage D. ACHD = adult congenital heart disease; other abbreviation as in Figure 1.

Figure 1 demonstrates median KCCQ scores compared across anatomic complexity groups. Compared to simple or moderate complexity, KCCQ scores were significantly lower in those with great anatomic complexity (76.6 vs 89.0; *P* = 0.015). KCCQ scores were also significantly lower when compared across ACHD physiological stage (98.0 in A, 72.6 in D, r = −0.42; *P* < 0.001; 95% CI: -0.58,-0.23) and is demonstrated in Figure 2. Patients with worse NYHA score were also noted to have lower KCCQ scores (NYHA I: 96.5, NYHA II: 80.8, NYHA III: 44.4, r = −0.59; *P* < 0.001 95% CI: -0.71,-0.43).

### Predictive validity

Within the overall cohort, 92 patients had at least 6 months of follow-up after completion of the KCCQ and were included in the predictive validity analysis. Median follow-up time was 16.3 months. Table 3 represents the patient and disease demographics within the predictive validity analysis stratified by KCCQ score group. Patients in the highest score group were significantly more likely to have higher NYHA functional class compared to the middle and lowest score groups (*P* < 0.001). Compared to the middle and highest score groups, patients scoring ≤50 were significantly more likely to have sinus and/or atrioventricular node dysfunction (*P* = 0.015). There were no significant differences between score groups for the remainder of patient characteristics.

**Table 3:** Patient and Disease Demographics of the Predictive Validity Analysis Stratified by KCCQ Score Group

	Overall (N = 92)	KCCQ Score Groups			
		76-100 (n = 55, 59.8%)	51-75 (n = 21, 22.8%)	0-50 (n = 16, 17.4%)	*P* Value
Age (y)	28.2 (20.6-43.2)	27.4 (19.8-38.4)	23.8 (21.5-42.0)	40.3 (25.8-45.0)	0.137
Female	37 (40.2%)	24 (43.6%)	7 (33.3%)	6 (37.5%)	0.694
Anatomic complexity					0.134
Simple or moderate	36 (39.1%)	26 (47.3%)	5 (23.8%)	5 (31.3%)	
Great complexity	56 (60.9%)	29 (52.7%)	16 (76.2%)	11 (68.8%)	
Morphologic systemic ventricle					0.055
Left	61 (66.3%)	42 (76.4%)	9 (42.9%)	10 (62.5%)	
Right	30 (32.6%)	12 (21.8%)	12 (57.1%)	6 (37.5%)	
Heterotaxy	8 (8.7%)	4 (7.3%)	3 (14.3%)	1 (6.3%)	0.581
Atrial arrhythmia	39 (42.4%)	19 (34.5%)	11 (52.4%)	9 (56.3%)	0.174
Ventricular arrhythmia	26 (28.3%)	16 (29.1%)	4 (19.1%)	6 (37.5%)	0.456
SA and/or AV node dysfunction	25 (27.2%)	11 (20.0%)	5 (23.8%)	9 (56.3%)	0.015
Systemic ventricular dysfunction ≥ moderate	20 (21.7%)	10 (18.2%)	3 (14.3%)	7 (43.8%)	0.059
Atrioventricular valve dysfunction ≥ moderate	12 (13.0%)	10 (18.2%)	2 (9.5%)	0 (0.0%)	0.142
NYHA functional class	(N = 79)	(n = 49, 62.0%)	(n = 15, 19.0%)	(n = 15, 19.0%)	<0.001
I	22 (27.8%)	21 (42.9%)	1 (6.7%)	0 (0.0%)	
II	40 (50.6%)	23 (46.9%)	11 (73.3%)	6 (40.0%)	
III	16 (20.3%)	5 (10.2%)	3 (20.0%)	8 (53.3%)	
IV	1 (1.3%)	0 (0.0%)	0 (0.0%)	1 (6.7%)	
ACHD physiological stage	(N = 81)	(n = 50, 61.7%)	(n = 16, 19.8%)	(n = 15, 18.5%)	0.063
A	3 (3.7%)	3 (6.0%)	0 (0.0%)	0 (0.0%)	
B	11 (13.6%)	10 (20.0%)	1 (6.2%)	0 (0.0%)	
C	41 (50.6%)	24 (48.0%)	11 (68.8%)	6 (40.0%)	
D	26 (32.1%)	13 (26.0%)	4 (25.0%)	9 (60.0%)	

Values are n (%) or median (IQR).

Tests used: chi-square and Kruskal-Wallis tests for categorical and continuous variables, respectively.

AV = atrioventricular; KCCQ = Kansas City Cardiomyopathy Questionnaire; SA = sinoatrial; other abbreviation as in Table 1.

Of the patients who met the outcomes of interest, 3 patients died, 24 had at least one hospitalization, and 22 interventional cardiac procedures were performed. Causes of death included multi-organ failure in the context of acute or chronic HF, an unexpected outpatient death (presumed sudden cardiac death), and in the postoperative setting shortly after heart/liver transplant. The most common reasons for hospitalization were HF (7), arrhythmia (5), and infection (5). Of the cardiac procedures performed, 7 were interventional cardiac catheterization, 7 were cardiac surgery, 5 were electrophysiological ablation, and 3 were permanent pacemaker placement. Two cardiac catheterizations and one cardiac surgery were performed after a separate initial cardiac procedure and were thus excluded from DP analysis. In addition to the 2 patients who died after hospitalization, the outpatient death was also included in the DH analysis. All deaths were included in the DP analysis. The total number of included events for DH and DP were 25 and 22, respectively.

**Figure 3:**
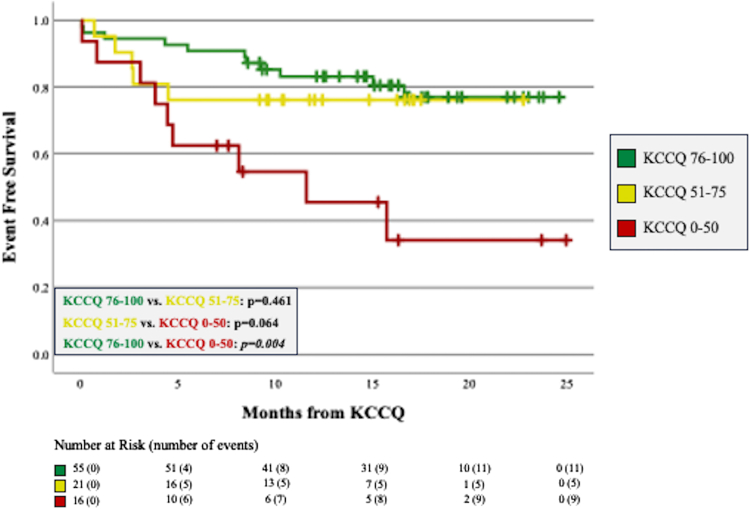
**Freedom From Death or Hospitalization Stratified by KCCQ Score Groups** Kaplan-Meier curves showing freedom from the outcome of death or all-cause hospitalization. Compared to patients scoring 76 to 100, those scoring ≤50 had significantly worse event-free survival. Abbreviation as in Figure 1.

**Figure 4:**
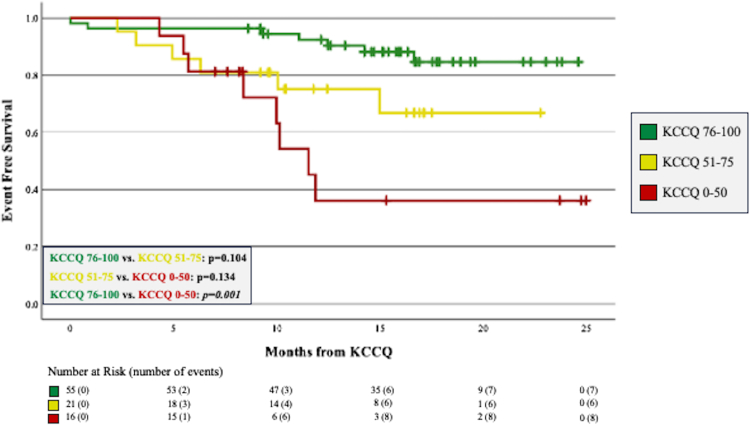
**Freedom From Death or Procedures Stratified by KCCQ Score Groups** Kaplan-Meier curves showing freedom from the outcome of death or interventional cardiac procedures including cardiac surgery, cardiac catheterization intervention, electrophysiological ablation, or placement of a permanent pacemaker. Patients scoring ≤50 had significantly worse event free survival compared to patients who scored 76 to 100. Abbreviation as in Figure 1.

Patients who experienced DH had a significantly lower KCCQ score compared to those who did not (66.0 vs 85.0; *P* = 0.002). Similarly, patients with DP scored significantly lower than those who did not meet this composite outcome (53.5 vs 85.0, *P* = 0.001). Event-free survival curves are shown for DH and DP in Figure 3, Figure 4, respectively. Compared to patients scoring 76 to 100, event-free survival was significantly worse for patients scoring ≤50 (*P* = 0.004 for DH, *P* = 0.001 for DP).

## Discussion

The KCCQ has previously been validated in the non-ACHD HF population,^8^ but its utility has yet to be directly studied in patients with ACHD. In this study, great anatomic complexity, advanced ACHD physiological stage, physician-reported HF, and advanced NYHA functional class were significantly associated with lower KCCQ scores in ambulatory ACHD patients, thereby demonstrating construct validity in the ACHD population. Additionally, ACHD patients who ultimately died, were hospitalized, or underwent an interventional cardiac procedure had median KCCQ scores that were substantially lower than those who did not experience those outcomes (Central Illustration). Freedom from DH and DP was significantly worse for patients scoring ≤50 on the KCCQ, thereby demonstrating predictive validity of the KCCQ in patients with ACHD. We believe these findings help to validate the use of the KCCQ in the ACHD population; thus, providing better evidence for clinicians and investigators to utilize this PRO tool in both ACHD patient care as well as in future research endeavors.

**Central Illustration:**
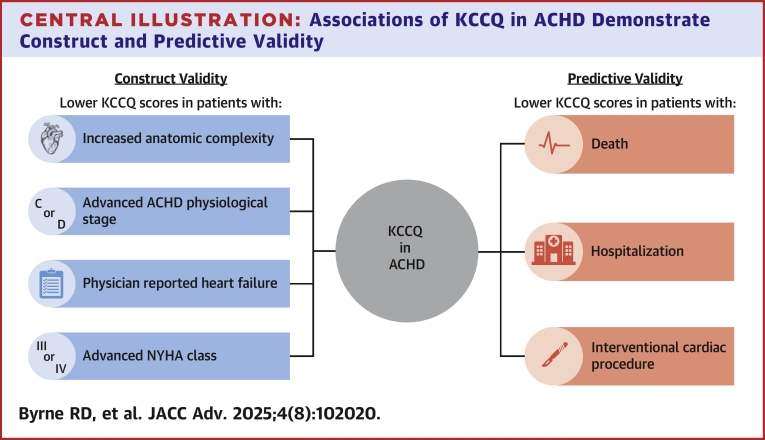
**Associations of KCCQ in ACHD Demonstrate Construct and Predictive Validity** In a single-center, cross-sectional study of ambulatory ACHD patients (129 KCCQs total, median age 29.0 years, 41.3% female), lower KCCQ scores were associated with great anatomic complexity, advanced ACHD physiological stage, physician-reported HF, and advanced NYHA functional class thereby demonstrating construct validity. To demonstrate predictive validity, a prospective cohort study of patients with at least 6 months follow-up was performed (N = 92, median follow-up 16.3 months). ACHD patients who ultimately died, were hospitalized, or underwent an interventional cardiac procedure had significantly lower KCCQ scores, demonstrating predictive validity. These findings provide an opportunity to utilize the KCCQ to assess health status in ACHD both clinically and in future scholarly work. Abbreviations as in Figures 1 and 2.

As the ACHD population grows, so does our understanding of the patient experience of living with congenital heart disease into adulthood. PROs improve this insight and have been an emergent area of interest within ACHD.^19^^,^^20^ The KCCQ provides not just an assessment of symptom burden and frequency, such as ACHD physiological stage or NYHA functional class similarly indicate, but it additionally offers understanding of other domains of a patient’s health status such as physical and social limitations. Incorporating PROs such as the KCCQ into the ACHD clinical evaluation invites inclusion of the patient’s voice alongside the physician’s clinical assessment and provides the clinician with important quality of life data that have prognostic implications as well.

### Construct validity

Lower KCCQ scores were significantly associated with all physician assessments of physiologic failure in our cohort. Given the mechanistic diversity of physiologic failure in ACHD, other key findings of our analysis provide added understanding of determinants of health status within the ACHD HF experience. Notably, atrial arrhythmia was associated with significantly lower KCCQ scores. Arrhythmia additionally accounted for the second most common cause of hospitalization in our cohort, behind HF. While HF remains a predominant final common pathway within ACHD, particularly for complex phenotypes,^1^^,^^2^^,^^21^ arrhythmia is also a frequent clinical feature.^22^^,^^23^ Often, HF and arrhythmia coexist,^2^^,^^24^ and each may be the cause or effect of a declining ACHD physiology. Thus, arrhythmia undoubtedly contributes to ACHD HF symptom experience and overall quality of life. The ACHD PRO, designed as a disease-specific PRO measure for patients with ACHD, acknowledges the significance of this clinical element within ACHD HF and emphasizes arrhythmia as a key domain for which it assesses.^19^ Whether caused by arrhythmia or a more traditional HF phenotype, a low KCCQ score can indicate the presence of a physiologic perturbation. In a population with considerable HF heterogeneity, it is then incumbent on the ACHD physician to pursue appropriate clinical investigation and determine the etiology.

Patients with a systemic right ventricle had significantly lower KCCQ scores compared to patients with a systemic left ventricle suggesting an overall worse health status for patients with transposition of the great arteries with atrial switch, congenitally corrected transposition of the great arteries without anatomic repair, and single ventricle patients with a morphologic right ventricle as their systemic ventricle. Patients with single ventricle physiology in our study had numerically lower, but not statistically different, KCCQ scores than patients with biventricular physiology. While others have reported KCCQ-12 scores to be lower in Fontan patients compared to patients with hemodynamically insignificant shunt lesions,^25^ our findings are likely influenced by greater anatomic complexity in our patients with biventricular circulation. Patients with hemodynamically insignificant shunting lesions would largely be categorized as simple anatomic complexity. In our study population, only 4 patients were categorized as such, with the greater majority of biventricular circulations being anatomies with moderate or great complexity. Furthermore, nearly 25% of our biventricular circulation patients were those with systemic right ventricle, likely driving down the median score for biventricular patients in our study.

Patients with lower systemic oxygen saturation had significantly lower KCCQ scores. Cyanosis in ACHD is often a surrogate marker of a deteriorating physiology, particularly in patients with single ventricle^26^ and can itself contribute to the symptom burden of ACHD HF. Systemic oxygen saturations can be noninvasively obtained in the ambulatory setting and, when abnormal, can provide additional suggestion that a physiology is failing.

Moderate or worse systemic ventricular dysfunction by echocardiogram was not associated with lower KCCQ scores in ACHD. While ACHD patients can and do develop HF related to systolic ventricular dysfunction,^2^ the absence of its association with lower KCCQ scores is notable for 2 reasons. The first is that this example highlights the proclivity for ACHD patients to adapt to their underlying physiologic limitations despite reduced functional capacity.^27^ ACHD patients may not experience a significant difference in symptoms in the setting of a nominal decrease in systolic function because important inherent circulatory inefficiencies are accommodated for in a depressed, baseline functional status. Likely, there is a threshold at which decreased systolic function does affect symptomatology which this study is underpowered to assess. Therefore, a low KCCQ score should all the more raise concern for a declining health status in an ACHD patient. The second key point is that this example provides yet another reminder that the phenotype of physiologic failure in ACHD often departs from what is traditionally seen in the non-ACHD HF population.^28^

### Predictive validity

ACHD patients who reached the endpoints of DH or DP had significantly lower KCCQ scores than those who did not experience these outcomes and the lowest scoring KCCQ group had significantly worse event-free survival. These findings demonstrate predictive validity of the KCCQ in ACHD. As expected, the indications for hospitalization and variety of endpoints met for interventional cardiac procedures match the etiologic diversity within ACHD HF. The survival curves for each composite outcome demonstrate early divergence with most outcomes met within the first year. In our study, within 12 months of scoring ≤50 on the KCCQ, approximately 50% of patients experienced death or hospitalization and nearly 2-thirds of patients at risk died or underwent an interventional cardiac procedure. This prognostic feature of a low KCCQ score can provide the ACHD physician with additional evidence by which to recommend a medication change or procedural intervention. Similarly, ACHD physicians should strongly consider referring patients with a low KCCQ score to an ACHD HF specialist given both the benefits of early referral^3^^,^^29^ and the previously recognized utility of the KCCQ in predicting advanced therapies in the non-ACHD population.^30^

### KCCQ in future ACHD research

The KCCQ is used extensively as an outcome measure in high-impact scholarly works in the non-ACHD population.^10^, ^11^, ^12^ We believe future ACHD outcomes studies have an opportunity to incorporate evaluation of the responsiveness of health status to interventions, similar to its use in the non-ACHD population. While we demonstrate construct and predictive validity of the KCCQ in ACHD patients in the present study, future investigation establishing reproducibility and responsiveness validity of the KCCQ within ACHD might help bolster its scholarly potential in this field.

### Study Limitations

Predictor variables such as NYHA functional class, reporting of HF as a patient diagnosis, and interpretation of ECHO findings such as ventricular function were determined at the discretion of the cardiologist, increasing the possibility of variation in these clinical assessments. Cardiac medications were not reported, though could have impacted KCCQ score in our patients, and should be the focus of future investigations. Furthermore, in this single-center study, institutional bias in our approach to pursuing interventional cardiac procedures as defined by this study may affect generalizability of the predictive validity of the KCCQ for this particular outcome variable.

Although the KCCQ was offered to all patients in our ACHD clinic, inherent bias exists within our study due to the nonuniformity of patients who ultimately completed the questionnaire. For instance, the KCCQ survey was only offered in English which may have been a barrier for non-English speakers. The majority of patients who completed a KCCQ did so in our ACHD HF clinic resulting in selection of a relatively sicker patient group than that of the general ACHD population. However, the KCCQ was originally intended for a HF population, and it is perhaps the ACHD HF cohort wherein the KCCQ is most clinically useful for ACHD patients.

Finally, our study’s population size is relatively small with limited outcome events, restricting our ability to perform multivariable analysis.

## Conclusions

In a single-center, ambulatory ACHD population, KCCQ scores were significantly lower for patients with great anatomic complexity, advanced ACHD physiological stage, physician-reported HF, and advanced NYHA functional class. ACHD patients who ultimately died, were hospitalized, or underwent an interventional cardiac procedure had significantly lower KCCQ scores and patients scoring ≤50 had significantly worse event-free survival. This study is the first to demonstrate construct and predictive validity of the KCCQ in the ACHD population and provides a basis for future use of KCCQ within ACHD in both a clinical and scholarly context.

Perspectives **COMPETENCY IN PATIENT CARE 1:** Utilization of the KCCQ in ACHD provides insight into a patient’s health status and adds to the overall clinical assessment of patients with ACHD HF.

**COMPETENCY IN PATIENT CARE 2:** A low KCCQ score alerts the ACHD physician to the likelihood of physiologic failure and provides an opportunity for medical and/or procedural intervention as well as referral for consideration of advanced therapies if needed.

**TRANSLATIONAL OUTLOOK 1:** Given the demonstration of construct and predictive validity of the KCCQ in ACHD, future ACHD investigators may consider its use as an outcome measure in their scholarly work.

**TRANSLATIONAL OUTLOOK 2:** Future investigation establishing reproducibility and responsiveness validity of the KCCQ within ACHD will help strengthen its clinical and scholarly potential in this field.

## Funding support and author disclosures

The authors have reported that they have no relationships relevant to the contents of this paper to disclose.
