# Supplementary material for: Construct and Predictive Validity of the Kansas City Cardiomyopathy Questionnaire in Adult Congenital Heart Disease
Source: JACC Adv. 2025 Jul 24;4(8):102020. doi: 10.1016/j.jacadv.2025.102020 (PMC12311496; doi:10.1016/j.jacadv.2025.102020)
Supplement: Supplemental Appendix [file mmc1.docx]

**Supplemental Figure 1**

Title: Cardiac Diagnoses Stratified by Anatomic Complexity

Caption: For construct validity statistical analysis, the overall cohort was stratified by anatomic complexity with the accompanying cardiac diagnoses. ASD = atrial septal defect. ccTGA = congenitally corrected transposition of the great arteries. CHD = congenital heart defect. DILV = double inlet left ventricle. DORV = double outlet right ventricle. D-TGA = dextro-transposition of the great arteries. HLHS = hypoplastic left heart syndrome. LV = left ventricle. RV = right ventricle. RV-PA = right ventricle to pulmonary artery. PA/IVS = pulmonary atresia with intact ventricular septum. VSD = ventricular septal defect.

**Supplemental Table 1: Domain Specific Scores in Key Clinical Features and Assessments of Physiologic Failure**

|  | **Symptom Burden** | | | **Symptom Frequency** | | | **Physical Limitations** | | | **Social Limitations** | | | **Quality of Life** | | |
| --- | --- | --- | --- | --- | --- | --- | --- | --- | --- | --- | --- | --- | --- | --- | --- |
|  | **Clinical Feature Present** | **Clinical Feature Absent** | **P** | **Clinical Feature Present** | **Clinical Feature Absent** | **P** | **Clinical Feature Present** | **Clinical Feature Absent** | **P** | **Clinical Feature Present** | **Clinical Feature Absent** | **P** | **Clinical Feature Present** | **Clinical Feature Absent** | **P** |
| **Patient Characteristics** |  | | |  | | |  | | |  | | |  | | |
| Systemic Right Ventricle | 73.3  (56.7-86.7) | 76.7  (60.0-93.3) | 0.572 | 85.0  (65.0-95.0) | 90.0  (65.0-100.0) | 0.378 | 80.0  (61.3-89.6) | 83.3  (68.8-95.8) | 0.208 | 75.0  (60.3-93.8) | 93.8  (70.3-100.0) | ***0.016*** | 66.7  (37.5-83.3) | 83.3  (50.0-91.7) | ***0.005*** |
| Single Ventricle | 73.3  (53.3-86.7) | 80.0  (60.0-93.3) | 0.095 | 82.5  (56.3-100) | 90.0  (70.0-100.0) | 0.422 | 77.1  (55.2-95.8) | 83.3  (69.8-95.8) | 0.104 | 81.3  (62.4-100.0) | 87.5  (64.6-100.0) | 0.285 | 77.9  (54.4-93.3) | 75.0  (50.0-91.7) | 0.315 |
| Heterotaxy | 86.7  (65.0-95.0) | 73.3  (60.0-93.3) | 0.308 | 100.0  (81.3-100.0) | 85.0  (65.0-100.0) | 0.190 | 83.3  (54.4-100.0) | 83.3  (66.7-95.8) | 0.925 | 87.5  (61.5-100.0) | 87.5  (62.5-100.0) | 0.774 | 70.8  (45.8-85.4) | 75.0  (50.0-91.7) | 0.568 |
| Atrial Arrhythmia | 66.7  (46.7-86.7) | 80.0  (66.7-95.0) | ***0.003*** | 80.0  (55.0-90.0) | 95.0  (80.0-100.0) | ***<0.001*** | 75.0  (60.0-87.5) | 87.5  (70.6-100.0) | ***0.018*** | 75.0  (62.3-93.8) | 93.8  (73.4-100.0) | ***0.007*** | 66.7  (33.3-83.3) | 83.3  (66.7-91.7) | ***<0.001*** |
| Ventricular Arrhythmia | 73.3  (50.0-93.3) | 73.3  (60.0-93.3) | 0.637 | 90.0  (60.0-100.0) | 85.0  (66.3-100.0) | 0.769 | 83.3  (67.7-95.8) | 83.3  (62.5-95.8) | 0.445 | 83.3  (62.5-100.0) | 87.5  (62.5-100.0) | 0.325 | 75.0  (37.5-91.7) | 75.0  (50.0-91.7) | 0.383 |
| History of Pacemaker | 60.0  (46.7-86.7) | 76.7  (60.0-93.3) | ***0.021*** | 80.0  (55.0-90.0) | 90.0  (75.0-100.0) | ***0.006*** | 70.8  (43.8-87.5) | 84.2  (70.2-95.8) | ***0.007*** | 62.5  (46.9-87.5) | 93.8  (75.0-100.0) | ***<0.001*** | 58.3  (25.0-83.3) | 79.2  (52.1-91.7) | ***0.004*** |
| **Non-Invasive Physiologic Performance** |  | | |  | | |  | | |  | | |  | | |
| Systemic Oxygen Saturation <95% | 60.0  (46.7-85.0) | 86.7  (66.7-100.0) | ***<0.001*** | 75.0  (45.0-98.8) | 90.0  (78.8-100.0) | ***0.005*** | 72.9  (50.0-90.6) | 91.7  (78.1-100.0) | ***<0.001*** | 75.0  (52.1-100.0) | 93.8  (75.0-100.0) | ***0.020*** | 75.0  (41.7-89.6) | 83.3  (56.3-91.7) | 0.054 |
| Systemic Ventricular  Dysfunction  ≥ Moderate | 80.0  (66.7-93.3) | 73.3  (53.3-93.3) | 0.301 | 90.0  (72.5-100.0) | 85.0  (60.0-100.0) | 0.493 | 85.0  (75.0-91.7) | 83.3  (62.5-100.0) | 0.979 | 87.5  (62.5-100.0) | 87.5  (62.5-100.0) | 0.946 | 75.0  (45.8-83.3) | 83.3  (50.0-91.7) | 0.17 |
| Systemic Atrioventricular Valve Dysfunction  ≥ Moderate | 80.0  (66.7-93.3) | 73.3  (53.3-93.3) | 0.511 | 85.0  (80.0-97.5) | 90.0  (60.0-100.0) | 0.951 | 87.5  (77.1-97.9) | 83.3  (62.5-95.8) | 0.327 | 87.5  (75.0-100.0) | 87.5  (62.5-100.0) | 0.627 | 83.3  (70.8-91.7) | 75.0  (50.0-91.7) | 0.630 |
| **Assessments of Physiologic Failure** |  | | |  | | |  | | |  | | |  | | |
| Physician Reported Heart Failure | 66.7  (46.7-86.7) | 86.7  (66.7-100.0) | ***<0.001*** | 80.0  (50.0-90.0) | 95.0  (80.0-100.0) | ***<0.001*** | 79.2  (54.2-91.7) | 87.5  (70.6-100.0) | ***0.002*** | 75.0  (50.0-100.0) | 93.8  (79.7-100.0) | ***0.002*** | 66.7  (33.3-83.3) | 83.3  (66.7-91.7) | ***<0.001*** |
| NYHA Class III or IV | 46.7  (36.7-63.3) | 86.7  (66.7-100.0) | ***<0.001*** | 50.0  (32.5-72.5) | 95.0  (80.0-100.0) | ***<0.001*** | 54.2  (37.5-75) | 87.5  (75.0-100.0) | ***<0.001*** | 50.0  (40.6-75.0) | 93.8  (75.0-100.0) | ***<0.001*** | 41.7  (20.8-75.0) | 83.3  (66.7-91.7) | ***<0.001*** |
| ACHD Physiological Stage C or D | 73.3  (51.7-93.3) | 93.3  (86.7-93.3) | ***0.021*** | 85.0  (60.0-100.0) | 100.0  (90.0-100.0) | ***0.018*** | 83.3  (61.9-95.8) | 95.8  (83.3-100.0) | ***0.023*** | 82.3  (61.3-100.0) | 100.0  (87.5-100.0) | ***0.001*** | 75.0  (41.7-91.7) | 91.7  (83.3-100.0) | ***<0.001*** |

^a^ Median (IQR).

^b^ Test Used: Mann Whitney U test.

^c^ ACHD = Adult congenital heart disease. NYHA = New York Heart Association

**Supplemental Table 2. Overall Patient and Disease Demographics Stratified by Anatomic Complexity with All KCCQ Surveys Including Duplicate Patients**

|  | **Overall** | **Simple or Moderate Complexity** | **Great Complexity** | **P** |
| --- | --- | --- | --- | --- |
| **Demographics** | **N=129** | **N=51 (39.5%)** | **N=78 (60.5%)** |  |
| **Age (years)** | 27.9 (20.5-42.2) | 26.1 (19.8-38.1) | 29.0 (22.5-44.5) | ***0.045*** |
| **Sex (female)** | 48 (37.2%) | 23 (45.1%) | 25 (32.1%) | 0.260 |
| **Systemic Ventricular Morphology** | | | | |
| **Biventricular Left** | 65 (50.4%) | 51 (100.0%) | 14 (17.9%) | ***<0.001*** |
| **Biventricular Right** | 22 (17.1%) | 0 (0%) | 22 (28.2%) |  |
| **Single Ventricle Left** | 28 (21.7%) | 0 (0.0%) | 28 (36.3%) |  |
| **Single Ventricle Right** | 13 (10.0%) | 0 (0.0%) | 13 (16.9%) |  |
| **Indeterminate Morphology** | 1 (0.8%) | 0 (0.0%) | 1 (1.3%) |  |
| **Atrial Arrhythmia** | 53 (41.1%) | 12 (23.5%) | 41 (52.6%) | ***<0.001*** |
| **Ventricular Arrhythmia** | 39 (30.2%) | 13 (25.5%) | 26 (33.3%) | 0.180 |
| **History of Pacemaker** | 31 (24.0%) | 4 (7.8%) | 27 (34.6%) | ***<0.001*** |
| **Heterotaxy** | 12 (9.3%) | 0 (0.0% | 12 (15.4%) | ***0.003*** |
| **Systemic Oxygen Saturation (%)** | 96.0 (91.8-98.0) | 97.0 (96.0-98.5) | 94.0 (90.0-96.5) | ***<0.001*** |
| **Systemic Ventricular  Dysfunction ≥ Moderate** | 31 (24.0%) | 13 (25.5%) | 18 (23.1%) | 0.966 |
| **Atrioventricular Valve  Dysfunction ≥ Moderate** | 15 (11.6%) | 2 (3.9%) | 13 (16.7%) | ***0.032*** |
|  |  |  |  |  |
| **NYHA Class** | **N=108** | **N=41 (78.8%)** | **N=67 (87.0%)** |  |
| **I** | 26 (24.1%) | 15 (36.6%) | 11 (16.4%) | 0.094 |
| **II** | 59 (54.6%) | 20 (48.8%) | 39 (58.2%) |  |
| **III** | 22 (20.4%) | 6 (14.6%) | 16 (23.9%) |  |
| **IV** | 1 (0.9%) | 0 (0.0%) | 1 (1.5%) |  |
|  |  |  |  |  |
| **ACHD Physiological Stage** | **N=109** | **N=42 (80.8%)** | **N=67 (87.0%)** |  |
| **A** | 4 (3.7%) | 1 (2.4%) | 3 (4.5%) | 0.466 |
| **B** | 13 (11.9%) | 7 (16.7%) | 6 (9.0%) |  |
| **C** | 51 (46.8%) | 21 (50.0%) | 30 (44.8%) |  |
| **D** | 41 (37.6%) | 13 (31.0%) | 28 (41.8%) |  |

^a^ N (%) or Median (IQR).

^b^ Test used: X^2^ used for categorical variables and Mann Whitney U test used for continuous variables.

^c^ ACHD = Adult congenital heart disease.

^d^ NYHA = New York Heart Association.

**Supplemental Table 3. Kansas City Cardiomyopathy Questionnaire Scores in Key ACHD Clinical Features and Assessments of Physiologic Failure with All KCCQ Surveys Including Duplicate Patients**

|  | **Yes** | **No** | **P** |
| --- | --- | --- | --- |
| **Patient Characteristics** | | | |
| Systemic Right Ventricle | 64.1 (51.1-89.7) | 83.6 (64.6-95.5) | ***0.018*** |
| Single Ventricle | 74.5 (53.7-91.7) | 83.5 (61.4-94.4) | 0.195 |
| Heterotaxy | 79.7 (52.7-95.4) | 81.0 (60.4-94.2) | 0.945 |
| Atrial Arrhythmia | 69.8 (51.5-85.0) | 86.2 (67.3-95.9) | ***<0.001*** |
| Ventricular Arrhythmia | 80.6 (45.8-93.1) | 81.2 (61.0-94.5) | 0.547 |
| History of Pacemaker | 57.4 (42.4-89.6) | 84.2 (63.3-95.2) | ***<0.001*** |
| **Non-Invasive Physiologic Performance** | | | |
| Systemic Oxygen Saturation <95% | 74.5 (45.6-85.6) | 87.9 (62.5-95.9) | ***0.010*** |
| Systemic Ventricular  Dysfunction ≥ Moderate | 80.1 (51.9-92.4) | 83.5 (59.1-94.9) | 0.592 |
| Systemic Atrioventricular Valve Dysfunction ≥ Moderate | 84.3 (77.1-94.4) | 81.9 (56.3-94.4) | 0.513 |
| **Assessments of Physiologic Failure** | | | |
| Physician Reported Heart Failure | 75.0 (44.5-88.3) | 86.9 (69.0-96.5) | ***<0.001*** |
| NYHA Class III or IV | 45.0 (35.4-76.5) | 87.2 (71.3-96.0) | ***<0.001*** |
| ACHD Physiological Stage C or D | 78.6 (51.3-92.2) | 95.4 (84.4-98.9) | ***<0.001*** |

^a^ Median (IQR).

^b^ Test used: Mann Whitney U test.

^c^ ACHD = Adult congenital heart disease.

^d^ NYHA = New York Heart Association.
